# Changes in hospitalizations for chronic respiratory diseases after two successive smoking bans in Spain

**DOI:** 10.1371/journal.pone.0177979

**Published:** 2017-05-24

**Authors:** Iñaki Galán, Lorena Simón, Elena Boldo, Cristina Ortiz, Rafael Fernández-Cuenca, Cristina Linares, María José Medrano, Roberto Pastor-Barriuso

**Affiliations:** 1 National Centre for Epidemiology, Instituto de Salud Carlos III (ISCIII), Madrid, Spain; 2 Department of Preventive Medicine and Public Health, School of Medicine, Universidad Autónoma de Madrid /IdiPAZ, Madrid, Spain; 3 CIBER for Epidemiology and Public Health (CIBERESP), Madrid, Spain; 4 Puerta de Hierro Biomedical Research Institute, Madrid, Spain; 5 National School of Public Health, Instituto de Salud Carlos III (ISCIII), Madrid, Spain; Istituti Clinici Scientifici Maugeri, ITALY

## Abstract

**Background:**

Existing evidence on the effects of smoke-free policies on respiratory diseases is scarce and inconclusive. Spain enacted two consecutive smoke-free regulations: a partial ban in 2006 and a comprehensive ban in 2011. We estimated their impact on hospital admissions via emergency departments for chronic obstructive pulmonary disease (COPD) and asthma.

**Methods:**

Data for COPD (ICD-9 490–492, 494–496) came from 2003–2012 hospital admission records from the fourteen largest provinces of Spain and from five provinces for asthma (ICD-9 493). We estimated changes in hospital admission rates within provinces using Poisson additive models adjusted for long-term linear trends and seasonality, day of the week, temperature, influenza, acute respiratory infections, and pollen counts (asthma models). We estimated immediate and gradual effects through segmented-linear models. The coefficients within each province were combined through random-effects multivariate meta-analytic models.

**Results:**

The partial ban was associated with a strong significant pooled immediate decline in COPD-related admission rates (14.7%, 95%CI: 5.0, 23.4), sustained over time with a one-year decrease of 13.6% (95%CI: 2.9, 23.1). The association was consistent across age and sex groups but stronger in less economically developed Spanish provinces. Asthma-related admission rates decreased by 7.4% (95%CI: 0.2, 14.2) immediately after the comprehensive ban was implemented, although the one-year decrease was sustained only among men (9.9%, 95%CI: 3.9, 15.6).

**Conclusions:**

The partial ban was associated with an immediate and sustained strong decline in COPD-related admissions, especially in less economically developed provinces. The comprehensive ban was related to an immediate decrease in asthma, sustained for the medium-term only among men.

## Introduction

Ample evidence links environmental exposure to second-hand tobacco smoke (SHS) to substantial harmful health effects, particularly to cardiovascular and respiratory systems [1,2]. Nevertheless, though SHS contains potent irritants which may lead to chronic inflammation and airway obstruction, current evidence is suggestive but not sufficient to infer a causal relationship between SHS exposure and the onset of asthma or chronic obstructive pulmonary disease (COPD) [1,2,3].

A wealth of research has evaluated the heath impact of the implementation of smoke-free laws. Several studies have examined outcomes based on changes in several respiratory symptoms among hospitality workers before and after the implementation of smoking bans. Based on questionnaire data [4–6] or pulmonary function tests [6] the evidence shows symptom reduction and improvements in pulmonary function parameters. Other studies have examined changes in the volume of hospital admissions with inconclusive results [7]; some show a reduction in hospitalizations for COPD [8–13], whereas others fail to detect any significant differences [14–16]. Similarly, most studies on asthma-related hospital admissions reported a reduction in hospitalizations [10,12,14,16–22], but others observed no significant changes [11,15].

In Spain smoke-free regulations have been introduced consecutively. The first law came into force on the 1^st^ of January of 2006 (partial ban) dictating a complete smoking ban at the workplace and a partial ban in hospitality venues (cafés, eateries, bars, and restaurants (bar and restaurants from now on) where partial restrictions applied according to venue size. Venues larger than 100m^2^ had to be 100% smoke-free or establish a physically separated smoking section (with its own ventilation system) not to exceed 30% of the total surface. Owners of smaller venues could continue allowing tobacco consumption, just as before the ban, or designate their venue as 100% smoke-free. This regulation achieved an important reduction in workplace-related SHS exposure but the impact was very modest in bars and restaurants [23].

To further reduce SHS exposure, a reform of the smoking legislation took effect on January 1^st^ of 2011 banning tobacco consumption in virtually all public spaces (comprehensive ban), including hospitality venues regardless of size. The new regulation eliminated all exceptions for hospitality venues drastically reducing SHS exposure in bars and restaurants [24]. Contrary to the industry´s fears, this ban had no negative impact on consumer expenditures in hospitality venues [25].

The main aim of the present work was to assess the joint association of two consecutive smoking bans and hospital admissions via emergency departments due to COPD and asthma using a large sample of the Spanish population.

## Material and methods

### Study population and data source

The study was conducted in the fourteen largest provinces of Spain for COPD, and in five provinces for asthma (see Table 1 for a list of the provinces included in each model). These provinces cover approximately 62% and 33% of the total population of Spain in 2011, respectively. The daily number of hospital admissions via emergency departments only (hospital admissions from now on) for COPD and asthma in each province from January 1^st^, 2003 through November 30, 2012 was obtained from the Hospital Discharge Records Database of the Spanish National Health System (CMBD-H for its acronym in Spanish), which receives discharge notifications from all public hospitals and private institutions within the consortium. We examined the number of daily hospital admissions for COPD as primary diagnosis (ICD-9 490–492, 494–496) in individuals 40 years or older, and for asthma (ICD-9 493) regardless of age. Daily population estimates in each province were calculated by linear interpolation from population figures at the beginning of each year provided by the Spanish National Institute of Statistics.

**Table 1:** Summary statistics for average daily hospital admissions and other population characteristics across the fourteen largest Spanish provinces, 2003–2012.

	Minimum	25th percentile	Median	75th percentile	Maximum
Average # of daily hospital admissions					
Chronic obstructive pulmonary disease*	1.3	3.7	5.6	8.1	33.1
Asthma†	0.9	1.7	5.3	8.8	9.6
Population size (thousands)	944	1,093	1,308	1,863	6,205
Population ≥ 65 years (%)	11.7	14.0	15.5	18.6	22.1
Latitude‡ (degrees north)	28.3	37.6	39.5	41.7	43.3
Human development index§	91.3	93.1	94.2	96.1	98.8
Population unemployed (%)	9.8	10.8	13.2	18.4	22.3
Population living in rural settings¶ (%)	4.5	8.3	12.9	18.0	29.6
Number of hospital beds per 1,000 population	2.7	3.1	3.5	3.8	4.8
Number of physicians per 1,000 population	3.4	4.0	4.5	5.2	6.4

* Records from the fourteen largest Spanish provinces (Alicante, Asturias, Illes Balears, Barcelona, Cádiz, A Coruña, Madrid, Málaga, Murcia, Las Palmas, Sevilla, Valencia, Bizkaia, and Zaragoza)

^†^ Restricted to the five provinces with pollen count information available (Asturias, Barcelona, Madrid, Murcia, and Zaragoza).

^‡^ Latitude of province centroid.

^§^ Composite index of life expectancy, education, and per capita income, with values close to 100 indicating higher levels of human development.

^¶^ Population living in municipalities with less than 10,000 inhabitants.

Daily temperatures were collected from the National Institute of Meteorology (http://www.aemet.es/), and influenza data came from the Spanish Influenza Sentinel Surveillance System (http://vgripe.isciii.es/gripe/inicio.do). The National Health System’s Hospital Discharge Records Database (CMBD) (https://www.msssi.gob.es/estadEstudios/estadisticas/cmbdhome.htm) provided the information on acute respiratory infections. Finally, the Spanish Society of Allergology and Clinical Immunology (SEAIC for its Spanish acronym; http://www.seaic.org/), the Spanish Aerobiology Network (REA for its Spanish acronym; http://www.uco.es/rea/), and the Asturias Regional Health Council (https://www.asturias.es/portal/site/astursalud/menuitem.2d7ff2df00b62567dbdfb51020688a0c/?vgnextoid=efb82617d02e7210VgnVCM10000098030a0aRCRD) provided the data on specific pollen counts. Relevant pollen count information was only available from five provinces in the country, limiting the asthma admissions-related analyses to these provinces.

This study was approved by the Institutional Review Board of the Spanish Institute of Health Carlos III (ISCIII for its acronym in Spanish). To guarantee patient confidentiality, clinical records data were anonymized and de-identified prior to analysis.

### Province-specific analyses

We adopted a two-stage hierarchical approach [26,27] to summarize the association of smoking bans with hospital admissions for chronic respiratory diseases using time-series data from multiple Spanish provinces. In the first stage, separate Poisson additive models allowing for overdispersion were fitted to daily data for each province to estimate changes in hospital admission rates for COPD and asthma after the implementation of the 2006 partial and the 2011 comprehensive smoking bans adjusted for seasonality, day of the week, temperature, influenza epidemic status, acute respiratory infection rates. Models for asthma-related admissions also included pollen counts. More specifically, the number of hospital admissions for each disease $Y_{t}^{p}$ on day *t* in province *p* was assumed to follow a Poisson distribution with mean $\lambda_{t}^{p}n_{t}^{p}$ and free dispersion parameter *ϕ*^*p*^; where $\lambda_{t}^{p}$ was the underlying admission rate and $n_{t}^{p}$ was the population at risk (individuals ≥ 40 years of age for COPD and of any age for asthma). To allow for both immediate and gradual effects of smoking bans on COPD- and asthma-related admissions, province-specific admission rates $\lambda_{t}^{p}$ were analyzed through the segmented log-linear model [28]

$$\begin{array}{ll} \text{log}\left(\lambda_{t}^{p}\right) & =\beta_{0}^{p}+\beta_{1}^{p}t+\left\{\beta_{2}^{p}+\beta_{3}^{p}\left(t-t_{1}\right)\right\}I\left(t\geq t_{1}\right)\\ & +\left\{\beta_{4}^{p}+\beta_{5}^{p}\left(t-t_{2}\right)\right\}I\left(t\geq t_{2}\right)+\text{confounders,} \end{array}$$

where *t* was the day indexed from day 1 (January 1^st^, 2003) to day 3,622 (November 30^th^, 2012), *t*_1_ and *t*_2_ were the days at which the partial and comprehensive smoking bans came into effect on January 1^st^, 2006, and January 2^nd^, 2011, respectively, and *I*(*t* ≥ *t*_1_) and *I*(*t* ≥ *t*_2_) were indicators for time periods after each smoking ban implementation. The coefficients $\beta_{0}^{p}$ and $\beta_{1}^{p}$ represented the intercept and slope of log admission rates for the 2003–2005 pre-ban period. The coefficient $\beta_{2}^{p}$ represented the immediate shift in log admission rates at the implementation of the partial ban on January 1^st^, 2006; whereas $\beta_{3}^{p}$ corresponded to the change in slope of log admission rates for the 2006–2010 partial ban period versus the pre-ban period. Similarly, the coefficient $\beta_{4}^{p}$ represented the immediate shift in log admission rates at the implementation of the comprehensive ban on January 2^nd^, 2011, whereas $\beta_{5}^{p}$ corresponded to the slope change in log admission rates for the 2011–2012 comprehensive ban period versus the previous 2006–2010 partial ban period. For comparison purposes, we also estimated the average daily admission rates for each calendar year by replacing the above segmented linear function of time with single-year indicators. This allowed the display of temporal trends in province-specific admission rates without imposing any particular functional form.

Based on previous work [29], all Poisson models were adjusted for the same confounders in all provinces and included harmonic terms for seasonality in admission rates, indicators for the day of the week, and smoothed functions of temperature, influenza epidemic status, and acute respiratory infection rates. Models for asthma admissions also included smoothed functions of pollen counts. Specifically, seasonal variations were modelled by using sine and cosine terms with both annual and semiannual periods to capture seasonal fluctuations of higher frequency than a simple sinusoidal curve [30]. The highest temperatures, influenza virus detection rates, number of hospital admissions for acute respiratory infections (matching the age range used for each outcome), and daily pollen counts for Olea, Poaceae, and Plantago (asthma models only) were averaged over the current day and the two previous days. The smoothed functions of these confounders were all penalized thin plate regression splines with a maximum of 3 degrees of freedom [31]. These splines adequately captured potential nonlinear relationships thus leaving little residual confounding. Sensitivity analyses with moving averages over longer lags of 0–4 days and smoothed functions with an increased maximum of 5 degrees of freedom provided similar control for confounding (data not shown). Further adjustment for secular trends in smoking prevalence did not substantially alter the results (data not shown).

To assess potential heterogeneity in the effect of smoking bans by sex and age, additional province-specific analyses were performed separately for men and women, as well as by age groups for COPD patients (40–64 years and ≥ 65 years) and asthma patients (< 15 years and ≥ 15 years). All Poisson additive models were fitted using penalized quasi-likelihood methods, as implemented in the function gam from the R package mgcv (R Foundation for Statistical Computing; Vienna, Austria. http://www.R-project.org/).

### Pooling results across provinces

In the second stage, the model coefficients estimated within each province were combined through random-effects multivariate meta-analytic models for correlated outcomes [30]. By doing so, we obtained a pooled estimate of the association between smoking bans and hospital admissions for the entire country, we quantified heterogeneity in the associations across provinces, and we identified province-level characteristics accounting for this heterogeneity. Specifically, the estimates ${\hat{\beta }}^{p}$ of the vector of true coefficients $\beta^{p}={\left(\beta_{1}^{p},\ldots ,\beta_{5}^{p}\right)}^{\prime }$ defining the province-specific linear trends in hospital admission rates during the pre-ban, partial ban, and comprehensive ban periods were assumed to follow a multivariate normal distribution with mean ***β***^*p*^ and estimated within-province covariance matrix **V**^*p*^. This normal approximation with accurately estimated variance was warranted by the large number of hospitalizations for each ban period in each province (Table 1). The true coefficients ***β***^*p*^ were then allowed to vary randomly across provinces according to a multivariate normal distribution with mean depending on province-level covariates$x^{p}={\left(1,x_{1}^{p},\ldots ,x_{k}^{p}\right)}^{\prime }$ and unknown unstructured between-province covariance matrix **∑**,

$${\hat{\beta }}^{p}|\beta^{p}∼N\left(\beta^{p},V^{p}\right),$$

$$\beta^{p}|\alpha ,\Sigma ∼N\left(X^{p}\alpha ,\Sigma \right),$$

where **X**^*p*^ was a block-diagonal matrix with the covariate vector **x**^*p*^′ repeated along the diagonal and ***α*** was a vector of parameters relating these covariates with each coefficient in ***β***^*p*^.

Initially, a random-effects multivariate meta-analysis [30] with no province-level covariates, other than the intercept term **x**^*p*^ = 1, was fitted with parameters ***α*** = (*α*_1_,…,*α*_5_)′ determining population-average linear trends for each ban period summarizing the association across all provinces. The pooled percent changes in hospital admission rates at the implementation of the partial ban and one year afterwards were estimated as 100{exp(*α*_2_)−1} and 100{exp(*α*_2_ + 365*α*_3_)−1}, respectively. Similarly, the pooled percent changes at the implementation of the comprehensive ban and one year afterwards were defined as 100{exp(*α*_4_)−1} and 100{exp(*α*_4_ + 365*α*_5_)−1}, respectively [26].

To explore potential differences in the association by province-level characteristics we included several variables as single second-stage covariates **x**^*p*^ = (1, *x*^*p*^)′ in separate random-effects multivariate meta-regression models [30]. These variables included: geographical latitude, human development index (a composite index of life expectancy, education, and per capita income), percentage of population unemployed, percentage of population living in rural settings, and number of hospital beds and physicians per 1,000 population in each province. Modifications of pooled ban effects by these province-level characteristics were contrasted through likelihood ratio tests comparing nested meta-regression models with and without the covariate.

The overall heterogeneity in the association across provinces, as well as the residual heterogeneity beyond that explained by province-level characteristics, were detected with the multivariate extension of the Cochran chi-squared test and quantified with the extended *I*^2^ statistic. The *I*^2^ statistic describes the proportion of total variation in estimates ${\hat{\beta }}^{p}$, i.e., it depicts the proportion of total variation in the derived province-specific associations due to heterogeneity [26,32]. The above random-effects multivariate meta-analysis and meta-regression models were fitted through maximum likelihood methods using the R package mvmeta (R Foundation for Statistical Computing).

## Results

**Fig 1:**
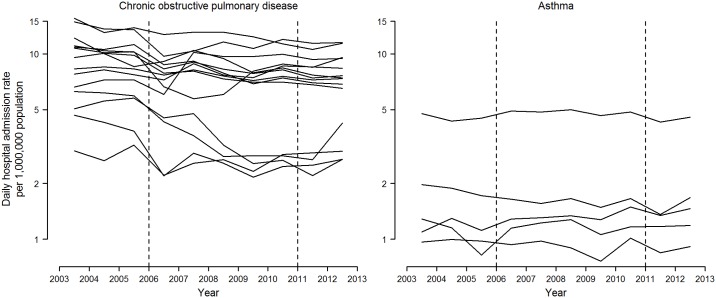
Daily hospital admission rates for chronic obstructive pulmonary disease and asthma by single calendar year in selected Spanish provinces, 2003–2012. Average daily admission rates for each calendar year were obtained from overdispersed Poisson additive models with single-year indicators and adjusted for seasonality, day of the week, temperature, influenza epidemics, acute respiratory infections, and pollen counts for asthma-related admissions (only available in Asturias, Barcelona, Madrid, Murcia, and Zaragoza). Rates refer to 1,000,000 population ≥ 18 years for cardiovascular diseases, ≥ 40 years for chronic obstructive pulmonary disease, and all ages for asthma. Vertical dashed lines represent the dates at which the partial and comprehensive smoking bans entered into force on January 1^st^, 2006, and January 2^nd^, 2011, respectively.

During the entire study period there were 431,797 hospital admissions for COPD in the fourteen largest Spanish provinces, as well as 95,411 hospital admissions for asthma in five provinces. By province, the average number of daily hospitalizations varied from 1.3 to 33.1 for COPD, and from 0.9 to 9.6 for asthma (Table 1). The province-specific daily hospital admission rates by single calendar year are displayed in Fig 1. Compared to asthma-related admissions, declines in COPD-related admission rates were more pronounced and heterogeneous across provinces, with an average decrease of 3.6% per year. The hospitalization rates for asthma remained fairly stable.

### Overall pooled associations of smoking bans on hospital admission rates

**Fig 2:**
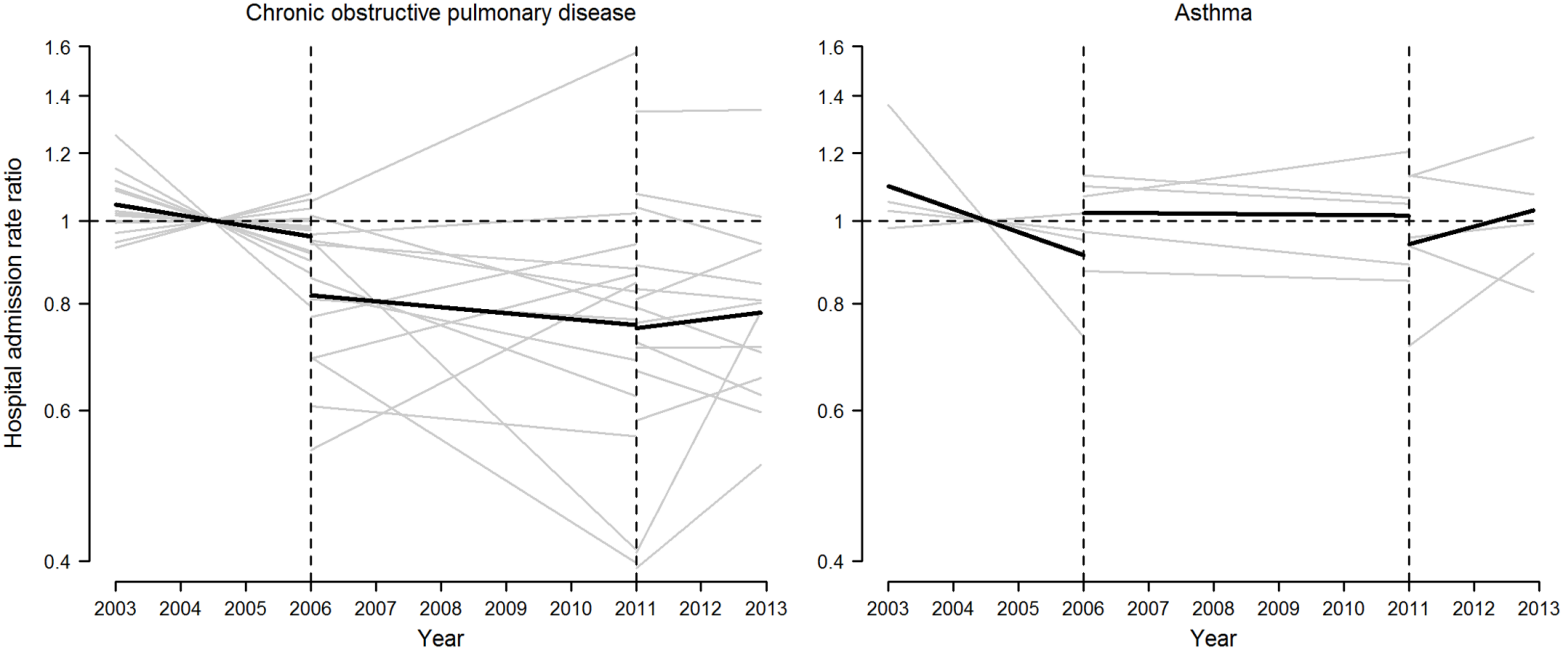
Population-average and province-specific segmented linear trends in hospital admission rate ratios for chronic obstructive pulmonary disease and asthma in selected Spanish provinces, 2003–2012. Province-specific segmented linear trends in hospital admission rate ratios (gray lines) were obtained from overdispersed Poisson additive models with distinct linear segments within the 2003–2005 pre-ban, 2006–2010 partial ban, and 2011–2012 comprehensive ban periods, and adjusted for seasonality, day of the week, temperature, influenza epidemics, acute respiratory infections, and pollen counts for asthma-related admissions (only available in Asturias, Barcelona, Madrid, Murcia, and Zaragoza). Pooled segmented linear trends (bold black lines) were obtained from random-effects multivariate meta-analyses on province-specific estimates of segmented regression coefficients. The reference time point (rate ratio = 1) was set at the midpoint pre-ban period (July 1^st^, 2004). Vertical dashed lines represent the dates at which the partial and comprehensive smoking bans entered into force on January 1^st^, 2006, and January 2^nd^, 2011, respectively.

Linear trends in hospital admission rates during the pre-ban, partial-ban, and comprehensive-ban periods varied widely across provinces (Fig 2). Over 90% of the variability in the estimated segmented linear trends was due to in-between province heterogeneity (*I*^2^ = 98.2% for COPD and 93.8% for asthma; all *P* values < 0.001). The pooled segmented linear trends across all provinces are displayed in Fig 2 and the corresponding pooled changes in hospital admission rates after the implementation of the partial and the comprehensive bans are summarized in Table 2.

**Table 2:** Pooled changes in hospital admission rates for chronic obstructive pulmonary disease and asthma immediately after and at the one-year mark of the implementation of the 2006 partial and 2011 comprehensive smoking bans, by sex and age group in selected Spanish provinces, 2003–2012*.

	2006 partial smoking ban†		2011 comprehensive smoking ban‡		*I*^2^§ (%)
	Immediate percent change (95% CI)	One-year percent change (95% CI)	Immediate percent change (95% CI)	One-year percent change (95% CI)	
**Chronic obstructive pulmonary disease**¶					
Overall	-14.7 (-23.4, -5.0)	-13.6 (-23.1, -2.9)	-0.9 (-4.4, 2.8)	3.0 (-5.4, 12.1)	98.2
Men	-15.6 (-23.5, -6.9)	-15.9 (-24.4, -6.5)	-2.1 (-5.9, 2.0)	2.5 (-6.1, 11.8)	97.9
Women	-16.8 (-28.3, -3.4)	-13.6 (-27.1, 2.4)	6.4 (-0.1, 13.2)	8.4 (-0.9, 18.5)	94.2
40–64 years	-17.3 (-26.7, -6.8)	-14.5 (-24.3, -3.4)	-2.8 (-9.6, 4.6)	-2.4 (-10.4, 6.4)	94.5
≥ 65 years	-14.8 (-23.5, -5.1)	-15.7 (-25.4, -4.6)	-1.4 (-5.3, 2.7)	3.4 (-5.3, 13.0)	97.7
Asthma**					
Overall	12.1 (-3.2, 29.8)	19.1 (-2.3, 45.1)	-7.4 (-14.2, -0.2)	-2.7 (-6.9, 1.6)	93.8
Men	14.8 (1.4, 30.0)	22.4 (11.0, 35.0)	-17.4 (-26.2, -7.6)	-9.9 (-15.6, -3.9)	89.3
Women	10.3 (-4.5, 27.4)	16.1 (-5.9, 43.3)	-3.5 (-10.2, 3.7)	-0.7 (-5.2, 3.9)	93.2
< 15 years	25.0 (-2.6, 60.4)	30.4 (4.9, 62.0)	-11.0 (-28.6, 11.1)	-1.1 (-14.3, 14.2)	93.1
≥ 15 years	8.6 (-4.9, 24.0)	18.3 (-3.3, 44.8)	-4.0 (-11.5, 4.0)	-3.2 (-7.9, 1.7)	91.2

* Province-specific segmented linear trends in hospital admission rates were first obtained from overdispersed Poisson additive models with distinct linear segments within the 2003–2005 pre-ban, 2006–2010 partial ban, and 2011–2012 comprehensive ban periods, and then combined across provinces through random-effects multivariate meta-analyses.

^†^ Pooled percent changes in hospital admission rates and their 95% confidence intervals (CIs) at the implementation and one-year following the partial ban compared with the projected trend from the pre-ban period.

^‡^ Pooled percent changes in hospital admission rates and their 95% CIs at the implementation and one year following the comprehensive ban compared with the projected trend from the previous partial ban period.

^§^ Overall *I*^2^ statistic for heterogeneity in ban effects across provinces.

^¶^ Pooled changes across the largest fourteen provinces (Alicante, Asturias, Illes Balears, Barcelona, Cádiz, A Coruña, Madrid, Málaga, Murcia, Las Palmas, Sevilla, Valencia, Bizkaia, and Zaragoza)

**Pooled changes across the five provinces with pollen count information available (Asturias, Barcelona, Madrid, Murcia, and Zaragoza).

The partial smoking ban was associated with a strong significant pooled immediate decline of 14.7% in COPD-related admission rates, which was sustained over time with a one-year 13.6% decrease. There was no subsequent effect of the comprehensive ban on COPD-related admission rates (immediate and one-year changes of -0.9% and 3.0%, respectively). After the partial ban, both sexes experienced the aforementioned strong pooled decline in COPD-related admissions (Table 2), though rates among women were partially offset by a subsequent increase (non-statistically significant (ns)) after the comprehensive ban (immediate and one-year increases of 6.4% and 8.4%, respectively). The sharp pooled declines in COPD-related admissions at the onset and the first year of the partial ban were observed both in middle-aged and elderly subjects (Table 2).

Asthma-related admission rates increased by 12.1% immediately after the partial ban, but decreased by 7.4% after the comprehensive ban, though these pooled estimates were highly imprecise. Also, the pooled rise in asthma-related admissions after the partial ban (immediate increases of 14.8% in men and 10.3% (ns) in women) was fully offset in men with the implementation of the comprehensive ban (an immediate decrease of 17.4%) but not in women (an immediate decrease of 3.5% (ns)).

### Pooled effects of smoking bans on hospital admission rates by province-level characteristics

Table 3 shows pooled changes in COPD-related admission rates by province-level characteristics. There were significant modifications of the magnitude of the partial smoking ban associations on COPD-related admission rates by human development index (*P* for effect modification = 0.04), percentage of population unemployed (*P* = 0.05), and percentage of rural population (*P* = 0.02). Both the immediate and one-year pooled declines in COPD-related admission rates reached 16.6% following the partial ban in provinces with a relatively low human development level (25th percentile). In provinces with a high unemployment rate (75th percentile), immediate and one-year rates declined 18.3% and 20.1%, respectively. Finally, in provinces with a relatively high percentage of rural population (75th percentile) rates decreased 19.2% and 18.4%, respectively. However, these factors contributed little to the high heterogeneity observed in COPD-related admission trends across provinces (all residual *I*^2^>97%). Effect modifications for asthma-related admissions were not explored due to the limited number of meta-analyzed provinces.

**Table 3:** Pooled changes in hospital admission rates for chronic obstructive pulmonary disease (COPD) immediately after and at the one-year mark of the implementation of the 2006 partial and 2011 comprehensive smoking bans by characteristics of the fourteen largest Spanish provinces, 2003–2012*.

25th and 75th percentiles of province-level characteristic	2006 partial smoking ban†		2011 comprehensive smoking ban‡		*p*-value for effect modification§	*I*^2^ for residual heterogeneity¶ (%)
	Immediate percent change (95% CI)	One-year percent change (95% CI)	Immediate percent change (95% CI)	One-year percent change (95% CI)		
Latitude (degrees north)					0.31	98.1
37.6	-15.5 (-24.8, -5.0)	-15.3 (-25.3, -4.0)	1.9 (-2.0, 6.0)	6.6 (-2.0, 15.9)		
41.7	-12.9 (-23.4, -1.0)	-10.6 (-22.0, 2.5)	-3.3 (-6.6, 0.2)	-1.2 (-9.8, 8.2)		
Human development index					0.04	97.4
93.1	-16.6 (-27.0, -4.8)	-16.6 (-27.6, -4.0)	0.7 (-4.1, 5.6)	10.9 (1.9, 20.6)		
96.1	-12.6 (-22.9, -0.9)	-10.3 (-21.5, 2.4)	-2.0 (-5.7, 1.9)	-3.2 (-10.5, 4.6)		
Population unemployed (%)					0.05	97.6
10.8	-11.0 (-22.4, 2.0)	-7.1 (-19.2, 6.9)	-3.0 (-7.1, 1.2)	-6.6 (-13.2, 0.5)		
18.4	-18.3 (-29.3, -5.5)	-20.1 (-31.2, -7.1)	2.2 (-3.4, 8.1)	15.5 (6.5, 25.4)		
Rural population (%)					0.02	98.2
8.3	-8.0 (-18.8, 4.1)	-6.5 (-18.2, 7.0)	0.9 (-3.6, 5.6)	6.6 (-4.2, 18.6)		
18.0	-19.2 (-27.8, -9.5)	-18.4 (-27.7, -7.9)	-2.2 (-6.2, 2.0)	0.5 (-8.8, 10.7)		
# of hospital beds per 1,000 population					0.32	98.1
3.1	-12.0 (-22.8, 0.2)	-13.5 (-25.1, -0.1)	0.0 (-4.5, 4.6)	8.8 (-0.8, 19.3)		
3.8	-16.6 (-25.9, -6.0)	-13.7 (-24.3, -1.5)	-1.4 (-5.2, 2.5)	-1.0 (-9.0, 7.7)		
# of physicians per 1,000 population					0.55	98.2
4.0	-15.4 (-26.2, -3.0)	-14.2 (-26.0, -0.5)	-0.7 (-5.4, 4.3)	7.3 (-3.1, 18.8)		
5.2	-13.9 (-24.3, -2.0)	-12.8 (-24.1, 0.2)	-1.0 (-5.1, 3.1)	-0.5 (-9.5, 9.5)		

* Pooled changes by province-level characteristics were obtained from separate random-effects multivariate meta-regression models relating province-specific estimates of segmented regression coefficients with their values of the corresponding characteristic as a single continuous covariate.

^†^ Pooled percent changes in hospital admission rates and their 95% confidence intervals (CIs)at the implementation and one year following the partial ban, compared with the projected trend from the pre-ban period, for the 25th and 75th percentiles of the province-level characteristic.

^‡^ Pooled percent changes in hospital admission rates and their 95% CIs at the implementation and one year following the comprehensive ban, compared with the projected trend from the previous partial ban period, for the 25th and 75th percentiles of the province-level characteristic.

^§^ Overall *P* value for modification of pooled ban effects by the corresponding province-level characteristic.

^¶^ Overall *I*^2^ statistic for residual heterogeneity in province-specific ban effects not explained by the corresponding province-level characteristic.

## Discussion

The implementation of the first smoking regulation, a partial ban, was associated with a strong immediate decline in COPD-related hospital admissions. This reduction persisted all throughout the regulation years experiencing only modest changes when the second smoking law, a comprehensive ban, came into force. The observed association is consistent across age and sex groups but stronger in less economically developed Spanish provinces.

Existing evidence of the impact of implementing smoke-free legislation on COPD-related hospital admissions remains scarce and results are inconsistent. Some studies report a decrease in admissions [8–13], whereas others failed to detect any changes [14–16]. There is a wide variation in strength of association reported in positive studies. The rate reduction detected in this work is similar to that reported by Vander Weg and colleagues [13] in the United States, and Dusemund and colleagues in Switzerland [6]. This reduction in COPD-related hospital admissions is larger than the reduction observed for heart ischemic diseases [33].

Our segmented-linear models allowed us to differentiate between the associations observed right after the law´s execution (immediate association) and those taking place over the medium-term. Thus, we were able to observe that the onset of the partial ban was associated with a rapid reduction in COPD-related hospital admissions. These results are consistent with the fast-acting harmful effects of SHS such as reduced pulmonary function and increased inflammatory processes, which have been observed even at low levels of exposure [34].

The current project´s pilot study evaluated the effect of the partial smoking ban on hospital admissions in the two largest Spanish cities, Madrid and Barcelona [29]. That study revealed that controlling for seasonality and related variables, such as flu and respiratory infection epidemics, had a moderate effect on hospital admission rate changes. In fact, in the time series, the 2005 COPD increase could be related to the strong flu epidemic of that same year [35] which may have brought forward the hospital admissions of the most vulnerable portion of the population, resulting, in turn, in a reduction in admissions in the following months, a pattern known as “harvesting effect.” Thus, we cannot rule out the possibility that the fall in admissions immediately following the ban´s implementation may be related to the previous year´s flu epidemic. However, the impact of this “harvesting effect” has proven small and limited in time (i.e., months) in relation to mortality [36]. Additionally, if admissions had been moved up, one would expect a subsequent gradual increase until reaching pre-2005 rates. In contrast, for the 5 years the partial ban was in place the slope representing number of admissions is parallel to the slope for the reference period (2003–2005), i.e., the effect remains consistent.

Regarding asthma-related hospital admissions, most authors report a decline in hospital admissions [10,12,16–21] and emergency department visits [14,22], although two other studies [11,15] failed to detect a favorable impact on hospitalizations. Unfortunately, our asthma-related results are not conclusive. In contrast with COPD-related results, the onset of the first ban is not related to a decrease in admissions but our data show a significant reduction in admissions immediately following the implementation of the comprehensive ban. However, this favorable change is maintained after one year only among men. Asthma is a highly heterogeneous condition presenting several phenotypes and widely differing epidemiological patterns by sex. Child population studies show that the effect of SHS exposure on respiratory function is far greater in boys than in girls, probably as a result of the faster bronchial development in girls [37]. Unfortunately, our study lacks the statistical power necessary for sex-stratified analyses for those under 15 years of age. Further research is needed in this area.

It is worth highlighting here the great variability in estimates across provinces for both COPD- and asthma-related admissions. This heterogeneity has been previously described by Tan and colleagues when comparing studies performed in different countries [33] as well asin a study on myocardial infarction morbidity and mortality based on a large sample of U.S. regions [38]. In this work, we were able to examine sources of variability only for COPD-related admissions given the small number of provinces included in the asthma-related analyses. A highly interesting find is the greater effect of the bans observed in provinces with lower levels of socioeconomic development, an understudied area of research. As far as we know, only one study carried out in Ireland has examined socioeconomic differentials in the impact of a national smoking ban on COPD mortality [39]. The authors detected the most pronounced COPD-related mortality reductions in the most disadvantaged tertile of the population. On the one hand, this finding is consistent with the knowledge that COPD is a disease strongly associated to socioeconomic status, being twice as prevalent among individuals of low socioeconomic level [40]. On the other hand, low socioeconomic level individuals are more likely to have greater SHS exposure [41] and, thus, are more likely to benefit from a reduction of SHS exposure in public spaces than other groups.

Nevertheless, residual variance remains very high, and it is likely that other variables, such as regional variability in compliance with the bans, may be related to differing rates of respiratory conditions-related hospital admissions. Estimates of police reports and sanctions by region varied widely from the time the partial ban was put in place, though compliance with the law cannot be compared due to the absence of official statistics [42].

Overall, more benefits on cardiovascular and respiratory diseases are observed in regions with comprehensive smoking laws banning smoking at the workplace as well as in bars and restaurants [33]. In fact, a U.S. study concluded that living in a community with a comprehensive smoke-free law reduced the risk of hospitalizations for COPD compared to living under a moderate-weak law [9]. However, current evidence includes important inconsistencies: three studies found reductions in COPD admissions in the context of partial laws [8,11,13], whereas three of the five studies evaluating comprehensive laws, failed to find a protective effect [14–16] and the other two showed an association [9,10]. Further, the one study evaluating asthma-related admissions under a partial ban failed to detect any effect [11]. Further research is required to identify how smoke-free policies may have differential effects on the disease burden of these two pathologies. For instance, given low rates of asthma-related hospital admissions compared to the high disease prevalence at the population level, primary care and emergency room visits might better capture the effects of smoking bans. These measures may be more relevant in the case of asthma because SHS exposure increases healthcare visits among non-severe cases [43].

However, despite both diseases being highly prevalent at the population level, not only COPD-related hospital admissions are far more common than asthma-related ones but one third of hospitalized patients is at risk of re-admission within 90 days, especially those with severe disease and high comorbidity [44]. Thus, COPD, as a condition sensitive enough to smoke-free policies, may be an suitable indicator to measure the effects of introducing non-comprehensive regulations. As far as we know only the Toronto (Canada) study by Naiman and colleagues [12] has evaluated the implementation of smoke-free policies in in three distinct phases. The first phase affected only the workplace environment including government offices; the second phase extended the ban to restaurants; and the final phase further extended the prohibition to bars. Reductions in hospital admission rates related to COPD and asthma were observed only after the second phase was implemented.

Our results should be interpreted in the context of the study’s limitations. The main one pertains to the ecological nature of the study which precludes the inference of causality. Additionally, simultaneous country-wide implementation of the regulations eliminated the possibility of a control group for comparisons.

Other limitations include the lack of information on patients´ smoking status on admission records. Further, despite adjusting for highly relevant covariates, changes in other factors not accounted for such as improvements in disease management during the period under study may have influenced our estimates, although any improvements are likely to have been too gradual to have any significant effect. Similarly, there might have been registry-related administrative factors affecting hospital admissions regarding these diseases. However, we are unaware of any such changes. Regarding asthma-related results for children, we only had the required data on specific pollen counts for five provinces, thus, limiting the statistical power necessary for sex-stratified analyses for those under 15 years of age. Larger samples are needed to further research in this area. Finally, meta-regression results are prone to low power issues and ecological bias when examining aggregate measures of individual-level characteristics.

Major strengths of this study include the analysis of a large sample including the fourteen largest Spanish regions and the availability of a large number of covariates modeled based on daily observations. The richness of these data allowed the creation of separate models for each province as well as by age and sex groups.

## Conclusions

The implementation of the first partial smoking ban is associated with a 14.7% decrease in COPD-related hospital admissions. This reduction remained steady and, basically, unaffected by the introduction of the comprehensive smoking ban. This association is consistent across sex and age groups and stronger in less economically developed provinces than in those with stronger economies. Unfortunately, asthma-related results are not conclusive. An immediate reduction in hospital admissions was observed after the implementation of the comprehensive ban. However, the reduction was sustained only for one year and only in men.

## References

[1] U.S. Surgeon General. The Health Consequences of Involuntary Exposure to Tobacco Smoke: A Report of the Surgeon General. Atlanta: US Department of Health and Human Services, Centers for Disease Control and Prevention, Coordinating Center for Health Promotion, National Center for Chronic Disease Prevention and Health Promotion, Office on Smoking and Health, 2006. 20669524

[2] U.S. Department of Health and Human Services. The Health Consequences of Smoking—50 Years of Progress: A Report of the Surgeon General. Atlanta, GA: U.S. Department of Health and Human Services, Centers for Disease Control and Prevention, National Center for Chronic Disease Prevention and Health Promotion, Office on Smoking and Health, 2014.

[3] Eisner MD, Anthonisen N, Coultas D, Kuenzli N, Perez-Padilla R, Postma D, et al An official American Thoracic Society public policy statement: Novel risk factors and the global burden of chronic obstructive pulmonary disease. Am J Respir Crit Care Med. 2010; 182:693–718. 10.1164/rccm.200811-1757ST 20802169

[4] Allwright S, Paul G, Greiner B, Mullally BJ, Pursell L, Kelly A, et al Legislation for smoke-free workplaces and health of bar workers in Ireland: before and after study. BMJ 2005; 331:1117 10.1136/bmj.38636.499225.55 16230313 PMC1283274

[5] Fernandez E, Fu M, Pascual JA, Lopez MJ, Perez-Rios M, Schiaffino A, et al Impact of the Spanish smoking law on exposure to second-hand smoke and respiratory health in hospitality workers: a cohort study. PLoS One. 2009; 4:e4244 10.1371/journal.pone.0004244 19165321 PMC2621339

[6] Frazer K, Callinan JE, McHugh J, van Baarsel S, Clarke A, Doherty K, et al Legislative smoking bans for reducing harms from secondhand smoke exposure, smoking prevalence and tobacco consumption. Cochrane Database Syst Rev. 2016;2:CD005992 10.1002/14651858.CD005992.pub3 26842828 PMC6486282

[7] Menzies D, Nair A, Williamson PA, Schembri S, Al Khairalla MZ, Barnes M, et al Respiratory symptoms, pulmonary function, and markers of inflammation among bar workers before and after a legislative ban on smoking in public places. JAMA. 2006; 296:1742–8. 10.1001/jama.296.14.1742 17032987

[8] Dusemund F, Baty F, Brutsche MH. Significant reduction of AECOPD hospitalisations after implementation of a public smoking ban in Graubünden, Switzerland. Tob Control. 2015; 24:404–7. 10.1136/tobaccocontrol-2013-051290 24500271

[9] Hahn EJ, Rayens MK, Adkins S, Simpson N, Frazier S, Mannino DM. Fewer hospitalizations for chronic obstructive pulmonary disease in communities with smoke-free public policies. Am J Public Health. 2014; 104:1059–65. 10.2105/AJPH.2014.301887 24825207 PMC4062034

[10] Head P, Jackson BE, Bae S, Cherry D. Hospital discharge rates before and after implementation of a city-wide smoking ban in a Texas city, 2004–2008. Prev Chronic Dis. 2012; 9:E179 10.5888/pcd9.120079 23270668 PMC3534134

[11] Humair JP, Garin N, Gerstel E, Carballo S, Carballo D, Keller PF, et al Acute respiratory and cardiovascular admissions after a public smoking ban in Geneva, Switzerland. PLoS One. 2014; 9:e90417 10.1371/journal.pone.0090417 24599156 PMC3944023

[12] Naiman A, Glazier RH, Moineddin R. Association of anti-smoking legislation with rates of hospital admission for cardiovascular and respiratory conditions. CMAJ. 2010; 182:761–7. 10.1503/cmaj.091130 20385737 PMC2871198

[13] Vander Weg MW, Rosenthal GE, Vaughan Sarrazin SM. Smoking bans linked to lower hospitalizations for heart attacks and lung disease among medicare beneficiaries. Health Aff (Millwood). 2012; 31:2699–2707. 23213154 10.1377/hlthaff.2011.0385

[14] Croghan IT, Ebbert JO, Hays JT, Schroeder DR, Chamberlain AM, Roger VL, et al Impact of a countywide smoke-free workplace law on emergency department visits for respiratory diseases: a retrospective cohort study. BMC Pulm Med. 2015; 15:6 10.1186/1471-2466-15-6 25608660 PMC4417313

[15] Gaudreau K, Sanford CJ, Cheverie C, McClure C. The effect of a smoking ban on hospitalization rates for cardiovascular and respiratory conditions in Prince Edward Island, Canada. PLoS One. 2013; 8:e56102 10.1371/journal.pone.0056102 23520450 PMC3592861

[16] Kent BD, Sulaiman I, Nicholson TT, Lane SJ, Moloney ED. Acute pulmonary admissions following implementation of a national workplace smoking ban. Chest. 2012; 142:673–9. 10.1378/chest.11-2757 22383660

[17] Herman PM, Walsh ME. Hospital Admissions for Acute Myocardial Infarction, Angina, Stroke, and Asthma After Implementation of Arizona's Comprehensive Statewide Smoking Ban. Am J Public Health. 2011; 101:491–6. 10.2105/AJPH.2009.179572 20466955 PMC3036684

[18] Landers G. The impact of smoke-free laws on asthma discharges: a multistate analysis. Am J Public Health. 2014; 104:e74–9. 10.2105/AJPH.2013.301697 PMC3935700 24328638

[19] Mackay D, Haw S, Ayres JG, Fischbacher C, Pell JP. Smoke-free legislation and hospitalizations for childhood asthma. N Engl J Med. 2010; 363:1139–45. 10.1056/NEJMoa1002861 20843248

[20] Millett C, Lee JT, Laverty AA, Glantz SA, Majeed A. Hospital admissions for childhood asthma after smoke-free legislation in England. Pediatrics. 2013; 131:e495–501. 10.1542/peds.2012-2592 23339216 PMC4528337

[21] Moraros J, Bird Y, Chen S, Buckingham R, Meltzer RS, Prapasiri S, et al The impact of the 2002 Delaware smoking ordinance on heart attack and asthma. Int J Environ Res Public Health. 2010; 7:4169–78. 10.3390/ijerph7124169 21318001 PMC3037047

[22] Rayens MK, Burkhart PV, Zhang M, Lee S, Moser DK, Mannino D, et al Reduction in asthma-related emergency department visits after implementation of a smoke-free law. J Allergy Clin Immunol. 2008; 122:537–41. 10.1016/j.jaci.2008.06.029 18692884

[23] Jimenez-Ruiz CA, Miranda JA, Hurt RD, Pinedo AR, Reina SS, Valero FC. Study of the impact of laws regulating tobacco consumption on the prevalence of passive smoking in Spain. Eur J Public Health. 2008; 18:622–5. 10.1093/eurpub/ckn066 18676987

[24] Lopez MJ, Fernandez E, Perez-Rios M, Martinez-Sanchez JM, Schiaffino A, Galan I, et al Impact of the 2011 Spanish smoking ban in hospitality venues: indoor secondhand smoke exposure and influence of outdoor smoking. Nicotine Tob Res. 2013; 15:992–6. 10.1093/ntr/nts218 23100458

[25] Caballero Hidalgo A, Pinilla Domínguez J. [Impact of new Spanish smoke-free legislation on the business activity of bars, cafes and restaurants]. Gac Sanit. 2014; 28:456–60. 10.1016/j.gaceta.2014.05.006 24950950

[26] Gasparrini A, Armstrong B, Kenward MG. Multivariate meta-analysis for non-linear and other multi-parameter associations. Stat Med. 2012; 31:3821–39. 10.1002/sim.5471 22807043 PMC3546395

[27] Burke DL, Ensor J, Riley RD. Meta-analysis using individual participant data: one-stage and two-stage approaches, and why they may differ. Stat Med 2017; 36: 855–75. 10.1002/sim.7141 27747915 PMC5297998

[28] Wagner AK, Soumerai SB, Zhang F, Ross-Degnan D. Segmented regression analysis of interrupted time series studies in medication use research. J Clin Pharm Ther. 2002; 27:299–309. 12174032 10.1046/j.1365-2710.2002.00430.x

[29] Galan I, Simon L, Flores V, Ortiz C, Fernandez-Cuenca R, Linares C, et al Assessing the effects of the Spanish partial smoking ban on cardiovascular and respiratory diseases: methodological issues. BMJ Open. 2015; 5:e008892 10.1136/bmjopen-2015-008892 26628524 PMC4679921

[30] Hunsberger S, Albert PS, Follmann DA, Suh E. Parametric and semiparametric approaches to testing for seasonal trend in serial count data. Biostatistics. 2002; 3:289–98. 10.1093/biostatistics/3.2.289 12933619

[31] Wood SN. Thin plate regression splines. J R Stat Soc Series B Stat Methodol. 2003; 65:95–114.

[32] Higgins JP, Thompson SG. Quantifying heterogeneity in a meta-analysis. Stat Med. 2002; 21:1539–58. 10.1002/sim.1186 12111919

[33] Tan CE, Glantz SA. Association between smoke-free legislation and hospitalizations for cardiac, cerebrovascular, and respiratory diseases: a meta-analysis. Circulation. 2012; 126:2177–83. 10.1161/CIRCULATIONAHA.112.121301 23109514 PMC3501404

[34] Flouris AD, Koutedakis Y. Immediate and short-term consequences of secondhand smoke exposure on the respiratory system. Curr Opin Pulm Med. 2011; 17:110–5. 10.1097/MCP.0b013e328343165d 21178628

[35] Larrauri A, Oliva J. Influenza surveillance in Spain. Spanish influenza surveillance sentinel network, 2005–2006 season [in Spanish]. Boletín Epidemiológico Semanal. 2006; 14:85–96. http://revista.isciii.es/index.php/bes/article/view/598/625.

[36] Goldstein E, Viboud C, Charu V, Lipsitch M. Improving the estimation of influenza-related mortality over a seasonal baseline. Epidemiology. 2012; 23:829–38. 10.1097/EDE.0b013e31826c2dda 22992574 PMC3516362

[37] Svanes C, Omenaas E, Jarvis D, Chinn S, Gulsvik A, Burney P. Parental smoking in childhood and adult obstructive lung disease: results from the European Community Respiratory Health Survey. Thorax. 2004; 59:295–302. 10.1136/thx.2003.009746 15047948 PMC1763798

[38] Shetty KD, DeLeire T, White C, Bhattacharya J. Changes in U.S. hospitalization and mortality rates following smoking bans. J Policy Anal Manage. 2010; 30:6–28. 21465828 10.1002/pam.20548

[39] Stallings-Smith S, Goodman P, Kabir Z, Clancy L, Zeka A. Socioeconomic differentials in the immediate mortality effects of the national Irish smoking ban. PLoS One. 2014; 9:e98617 10.1371/journal.pone.0098617 24887027 PMC4041857

[40] Gershon AS, Dolmage TE, Stephenson A, Jackson B. Chronic obstructive pulmonary disease and socioeconomic status: a systematic review. COPD. 2012; 9:216–26. 10.3109/15412555.2011.648030 22497534

[41] Gan WQ, Mannino DM, Jemal A. Socioeconomic disparities in secondhand smoke exposure among US never-smoking adults: the National Health and Nutrition Examination Survey 1988–2010. Tob Control. 2015; 24:568–73. 10.1136/tobaccocontrol-2014-051660 25015370

[42] Spanish Society of Epidemiology. Assessing the impact of the Spanish smoking law [in Spanish]. http://www.seepidemiologia.es/monografia.pdf.

[43] Comhair SA, Gaston BM, Ricci KS, Hammel J, Dweik RA, Teague WG, et al Detrimental effects of environmental tobacco smoke in relation to asthma severity. PLoS One. 2011; 6:e18574 10.1371/journal.pone.0018574 21572527 PMC3087715

[44] Hartl S, Lopez-Campos JL, Pozo-Rodriguez F, Castro-Acosta A, Studnicka M, Kaiser B, et al Risk of death and readmission of hospital-admitted COPD exacerbations: European COPD Audit. Eur Respir J. 2016; 47:113–21. 10.1183/13993003.01391-2014 26493806

